# Prominent Plasmacytosis Following Intravenous Immunoglobulin Correlates with Clinical Improvement in Guillain-Barré Syndrome

**DOI:** 10.1371/journal.pone.0002109

**Published:** 2008-05-07

**Authors:** Izumi Mori, Christophe Parizot, Karim Dorgham, Sophie Demeret, Zahir Amoura, Francis Bolgert, Guy Gorochov

**Affiliations:** 1 Institut National de la Santé et de la Recherche Médicale (INSERM) U543, Paris, France; 2 Laboratoire d'Immunologie Cellulaire et Tissulaire, Assistance Publique - Hôpitaux de Paris (AP-HP) Hôpital Pitié-Salpêtrière, Paris, France; 3 Department of Neurology, Assistance Publique - Hôpitaux de Paris (AP-HP) Hôpital Pitié-Salpêtrière, Paris, France; 4 Department of Internal Medicine, Assistance Publique - Hôpitaux de Paris (AP-HP) Hôpital Pitié-Salpêtrière, Paris, France; 5 Université Pierre et Marie Curie-Paris 6 (UPMC), Paris, France; Beijing Institute of Infectious Diseases, China

## Abstract

**Background:**

High doses of pooled polyclonal IgG are commonly used to treat numerous autoimmune diseases. Their mode of action nevertheless remains only partially explained. At the same time, until now, no early biological marker has been able to predict their efficacy.

**Methodology/Principal Findings:**

In a first pilot retrospective analysis, we reviewed white blood cell counts and blood smears in consecutive patients with autoimmune disease (n = 202) and non-autoimmune disease (n = 104). Autoimmune patients received either intravenous immunoglobulin (IVIg, n = 103), plasma exchange (n = 78) or no specific treatment (n = 21). We then prospectively monitored consecutive autoimmune patients with IVIg injection (n = 67), or without any specific treatment (n = 10) using the same routine laboratory tests, as well as flow cytometry. Both retrospective and prospective analyses identified large plasma-cell mobilization exclusively in IVIg-treated autoimmune patients 7 days after initiation of treatment. The majority of IVIg-mobilized plasma cells were immature HLA-DR^high^/CD138^low^/CXCR4^low^ plasma cells expressing intracellular immunoglobulin G which were neither IVIg- nor human IgG-specific. Importantly, we found a strong negative correlation between the absolute number of IVIg-mobilized plasma cells and time to improve neurological function in both retrospective and prospective studies of Guillain-Barré syndrome (GBS), (r = −0.52, p = 0.0031, n = 30, r = −0.47, p = 0.0028, n = 40, respectively).

**Conclusions/Significance:**

IVIg promotes immature plasma-cell mobilization in patients with GBS, chronic inflammatory demyelinating polyneuropathy, myasthenia gravis and inflammatory myopathy. Prominent day 7 plasma-cell mobilization is a favourable prognostic marker in patients with GBS receiving IVIg treatment.

## Introduction

Polyclonal IgG pooled from the serum of thousands of donors is widely used not only to confer passive protection to immune deficient patients but also as an anti-inflammatory agent [1]. Intravenous immunoglobulin (IVIg) therapy is approved by Food and Drug Administration for the treatment of immune thrombocytopenic purpura, Kawasaki disease, primary immunodeficiency, bone marrow transplantation, chronic B-cell lymphocytic leukemia, and pediatric HIV infection [2]. Off label use is common in several autoimmune conditions such as Guillain-Barré syndrome (GBS), chronic inflammatory demyelinating polyneuropathy (CIDP), myasthenia gravis (MG), inflammatory myopathy (IM), and multiple sclerosis, making it a major drug expenditure item [2]. Until now, there has existed no biological marker which may be used to evaluate the efficacy of this treatment. The lack of a biological marker is particularly troublesome when trying to evaluate the efficacy of IVIg during the course of chronic autoimmune diseases such as CIDP, MG and IM.

GBS is an autoimmune polyneuropathy, characterized by precedent infection and acutely progressive motor weakness. GBS affects 0.4–4.0 cases per 100,000 per year, and represents the most common cause of acute neuromuscular paralysis [3]. Older age, preceding gastro-intestinal infection, and rapid onset of severe motor weakness have been demonstrated to be adverse prognostic factors [4], [5]. Although IVIg has been shown to hasten the recovery of neurological function as efficiently as plasma exchange (PE), the mortality remains 5–10% in GBS [6], [7]. Until now, it has been impossible to predict which patient will benefit from a single course of IVIg, and which will need a more individualized treatment.

We report here that IVIg induces a peripheral mobilization of plasma cells in GBS, CIDP, MG and IM patients 7 days after initiation of treatment. Our most remarkable finding is that prominent IVIg-mobilized plasmacytosis correlates with faster recovery of neurological function in patients with GBS.

## Materials and Methods

### Objectives

The mode of action of IVIg remains only partially explained. We aimed to identify a biological marker to predict IVIg efficacy in autoimmune diseases.

### Participants

Fifty consecutive patients with GBS (M/F 31/19, median age 57, range 15–84), hospitalized in our institution between June 2004 and June 2007, were prospectively recruited. Three control autoimmune disease groups consisted of consecutive patients with CIDP (12/2, 52, 30–73), MG (4/3, 70, 19–94), and IM (2/4, 30, 23–57). Only GBS, CIDP, and MG patients were naïve of any previous immunomodulation therapy. Healthy bone marrow donors (2 men, 26, 32 years) and healthy controls (10/12, 39, 22–62) were enrolled in order to study medullary or circulating plasma cells.

Medical records of 157 GBS patients (91/66, 47, 15–83), referred to our institution between April 1990 and May 2004, were reviewed. We also analyzed medical records of consecutive autoimmune patients treated with IVIg (myasthenic respiratory crisis, 16/22, 58, 18–94, CIDP, 3/4, 55, 30–81) and consecutive non-autoimmune patients without immunomodulatory treatments (61/43, 53, 19–92, encephalitis n = 30, cerebral abscess n = 2, Lyme disease n = 1, botulism n = 1, status epilepticus n = 35, cerebral vascular disease n = 25, brain contusion n = 2, amyotrophic lateral sclerosis n = 8), admitted at our institution's neurological intensive care unit (ICU) for more than 14 days between April 1997 and May 2004.

All patients satisfied clinical diagnostic criteria of GBS [8], CIDP [9], MG [10] and IM [11]. We excluded GBS patients with serious pre-existing disease (lymphoma n = 1, colon cancer n = 2, cholangiocarcinome n = 1, hepatic abscess n = 1, toxoplasmosis n = 1).

### Ethics

The study was performed according to the Helsinki declaration. The ethics committee of Pitié-Salpêtrière Hospital approved the study, and signed informed consent was obtained from all patients participating in the prospective study.

### Assessments

The same senior neurologist (F.B.) examined all GBS patients and assessed GBS disability-grade [12]. GBS disability-grades were: 0 = healthy, 1 = minor symptoms and capable of running; 2 = able to walk 5 m or more without assistance but unable to run; 3 = able to walk 5 m across an open space with help; 4 = chairbound/bedbound: unable to walk as in 3; 5 = requiring assisted ventilation for at least part of the day; 6 = dead. We evaluated treatment efficacy by time to one GBS disability-grade improvement, capacity to walk independently at 2 months, and the need for and duration of artificial ventilation time.

### Treatments

All non-ambulant GBS patients were treated on day of admission (day 0). PE was the first line treatment between April 1990 and April 1997, and IVIg (0.4 g/kg/day, Tegeline, LFB laboratories, Les Ulis, France) has been the preferred treatment since then. PE-treated GBS patients with more than 4 disability-grade received six 50 mL/kg PE (n = 61), while patients with 3 disability-grade received only three PE courses (n = 17). GBS patients received IVIg for 5 days if their disability-grade was at least 4 (n = 81), or 3 days for grade 3 (n = 17). All CIDP patients in the retrospective study showed respiratory failure (n = 7), and therefore received IVIg for 4 days and oral prednisolone (1 mg/kg/day), while CIDP patients in the prospective study received IVIg only (n = 14). All MG patients (n = 45) received IVIg for 4 days and oral prednisolone (1 mg/kg/day). All IM patients (n = 6) received IVIg for 4 days after receiving intravenous pulse methylprednisolone (1 g/day) for 3 days.

### Routine enumeration of plasma cells

White blood cell count with 5 differentials was carried out on a XE2100 (SYXMEX) hematology analyzer, and plasma cell count was confirmed by blood smear exam after May Grunwald Giemsa staining.

### Cytometric analysis

Whole blood was collected at admission, and every 7 days during 28 days in the prospective population. Briefly, 200 µl of heparinized whole blood was incubated for 20 minutes at room temperature with various combinations of monoclonal antibodies. The following antibodies were used: with anti-CD19 allophycocyanin mAb (clone HIB19), anti-CD27 phycoerythrin mAb (clone M-T271), anti-CD27 fluorescein isothiocyanate (FITC) mAb (clone M-T271), anti-CD45 FITC (clone 2D1), anti-CD138 peridinin chlorophyll protein-cyanin 5.5 mAb (clone MI15), anti-CXCR4 phycoerythrin mAb (clone 12G5), anti-HLA-DR phycoerythrin-cyanin 7 mAb (clone L243), anti-CD38 FITC mAb (clone HIT2), anti-IgG FITC mAb (clone G18-145), anti-IgM FITC mAb (clone G20-127) (all from Becton Dickson; San Diego; CA) and human IgG FITC (Sigma; St. Louis; USA). IVIg FITC was prepared by incubation of 20 µl of 5 mg/ml FITC (Sigma) and 500 µl of 2 mg/ml IVIg (LFB) solutions for 2 hours. Unbound FITC was removed by dialysis against 0.1 M Tris-Cl, 0.2 M NaCl (Sigma) pH 7.4 over 2 days. For intracellular staining, cells were fixed with 1% paraformaldehyde (Sigma) for 20 minutes at room temperature after surface staining, and then permeabilized with 0.1% saponin (Sigma) before intracellular staining. Analyses were performed using a FACS Calibur and CellQuest Pro software (Becton Dickson). Absolute numbers of various CD19^+^CD27^high^ cell subsets were calculated per µl of blood, based on the frequencies of CD45^+^CD19^+^CD27^high^ cells among total CD45^+^ peripheral blood mononuclear cells (PBMCs).

### Statistical analysis

Proportions of patients between groups were compared using Fisher exact test. Mean values between two groups were compared by use of Mann-Whitney U test. Correlation was analyzed using Spearman test. Time to one disability-grade improvement comparison between two groups was performed using the log-rank test. In all statistical analysis, p values <0.05 were considered as statistically significant. Data were analyzed using StatView^®^ (SAS Institute, Cary, NC, USA).

## Results

### Demonstration of IVIg-induced plasmacytosis through retrospective analysis of autoimmune patients

**Figure 1:**
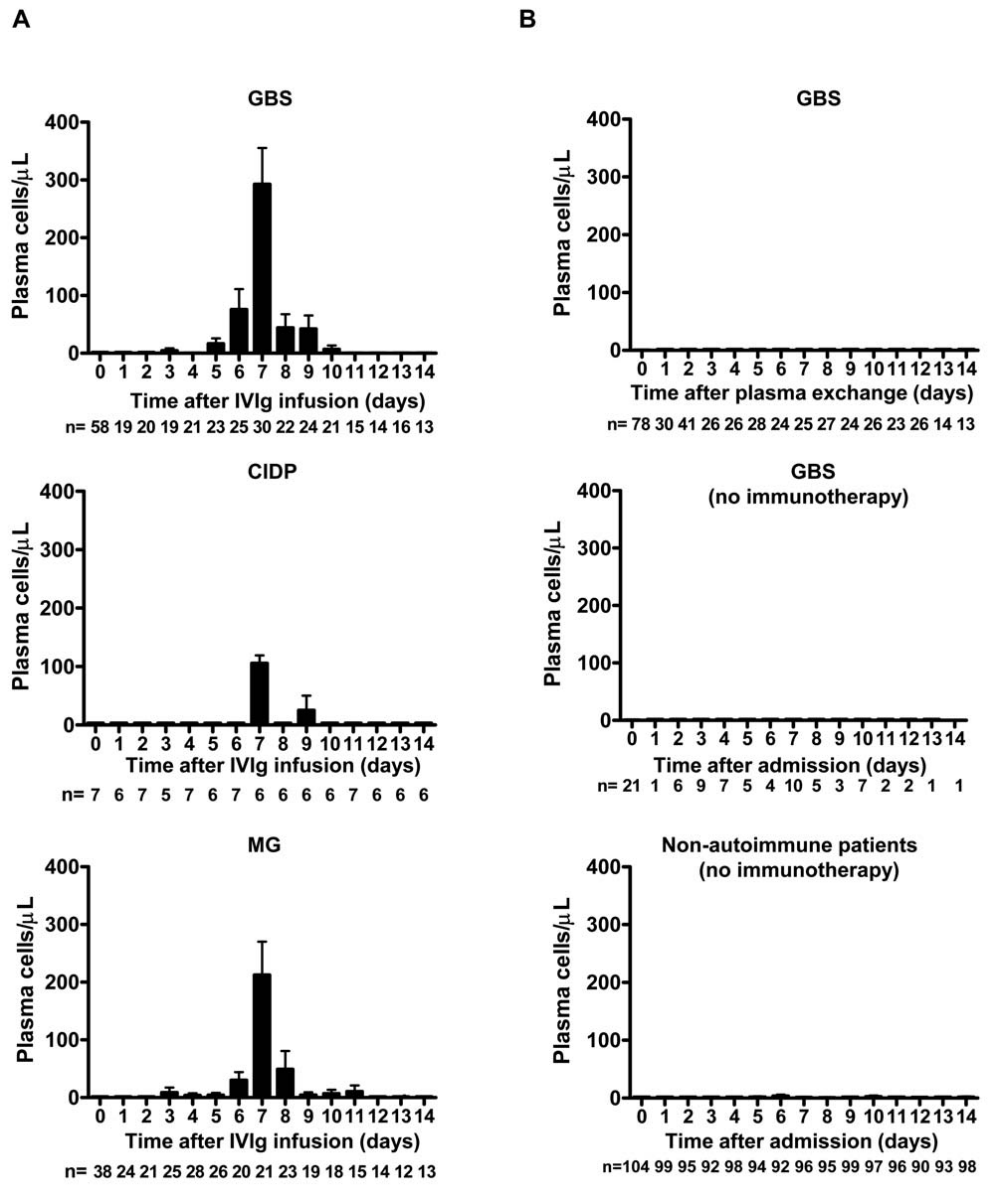
IVIg-related peripheral plasmacytosis peaks on day 7 of treatment in retrospective study. (A) Mean absolute number of plasma cells in IVIg-treated GBS (n = 58, upper panel), CIDP (n = 7, middle panel), and MG (n = 38, lower panel) patients. Severely disabled GBS patients (disability grade 3 or more) received IVIg only, whereas CIDP and MG patients received IVIg and steroids at admission (day0). (B) Mean plasma cell counts in non-IVIg-treated patients over a 14-day period following admission. Severely disabled GBS patients (n = 78, upper panel) were treated with plasma exchange at admission (day0). Moderately disabled GBS patients (disability grade 2 or less, n = 21, middle panel) and non-autoimmune patients (n = 104, lower panel) did not receive any immunotherapy during admission. At each time point, the number of patients with recorded white blood cell count is indicated. Data are mean ± s.e.m.

GBS represents a particularly attractive study model of autoimmunity insofar as it represents an acutely progressive polyneuropathy. A precipitating infection is frequently suspected, and the date of onset of neurological symptom is known. It therefore seemed to us particularly interesting to study in those patients the variations of the different leukocyte subpopulations. We detected a very large population of circulating plasma cells in numerous GBS afflicted patients treated with IVIg. A retrospective analysis allowed us to determine that this plasmacytosis was maximal on the 7^th^ day after initiating treatment (283 ± 343 cells/µl, n = 30, mean ± SD; Figure 1A upper panel). This effect is likewise found in patients treated for CIDP or for MG (107 ± 31 cells/µl, n = 6, 223 ± 272 cells/µl, n = 21, respectively; Figure 1A middle and lower panels). Therefore, this anomaly does not seem linked to a particular pathologic condition, but rather to IVIg treatment.

In order to confirm an association between IVIg and the observed plasmacytosis, we analyzed GBS patients having received PE only. We ensured that treatments with PE (n = 78) or IVIg (n = 58) were administered in isolation of other therapies, notably the absence of associated steroids. Furthermore, the two groups were homogeneous in terms of prognostic factors, including age (*p* = 0.98), previous diarrhoea (*p* = 0.50) and intervals from disease onset to treatment initiation (*p* = 0.09). We also included a third group of GBS patients (n = 21) that received no specific treatment whatsoever over the course of their hospitalization, however this group would obviously represent patients who were less severely afflicted, compared with IVIg-treated patients (*p* = 0.0008). In all these groups, we examined plasma-cell levels during a 14-day period following hospitalization. Peripheral blood plasmacytosis was not detected in patients treated with PE or in those without specific treatment (Figure 1B upper and middle panels). Importantly, the IVIg- and PE-treatment groups were homogeneous in terms of disability-grade at disease nadir (*p* = 0.29) and rate of improvement of neurological function (*p* = 0.84).

Although peripheral blood plasmacytosis was not detected in patients treated with PE, we were still concerned with the fact that this anomaly could simply reflect common infectious complications, and addressed this issue in two ways. Firstly, we verified that mean maximal plasma cell counts were not more elevated in IVIg-treated GBS patients with recorded infectious complications (pneumonia n = 24, septicaemia n = 1) than in the others during a 60-day period following hospitalization (278 ± 382 cells/µl, n = 25 vs 307 ± 410 cells/µl, n = 33, *p* = 0.33, data not shown). Secondly, we reviewed medical records of consecutive non-autoimmune ICU-hospitalized patients who never received immunomodulation therapy but who were exposed to the same infectious hazards. In this non-autoimmune group, plasmacytosis was rarely detected (Figure 1B, lower panel), and mean plasma cell counts 7 days after admission (1.8 ± 17 cells/µl, n = 96) were significantly less elevated than in IVIg-treated patients (GBS: 283 ± 343 cells/µl, n = 30, *p*<0.0001, CIDP: 107 ± 31 cells/µl, n = 6, *p*<0.0001, MG: 223 ± 272 cells/µl, n = 21, *p*<0.0001, data not shown). Importantly, the presence of plasmacytosis in non-autoimmune patients was always associated with sepsis, and sepsis-related plasmacytosis never exceeded 200 cells/µl during the 14-day hospitalization (54–187 cells/µl, n = 9). Altogether, it therefore seems reasonable to conclude that a prominent plasmacytosis exceeding 200 cells/µl 7 days after treatment was IVIg-induced.

### Confirmation of IVIg-induced plasmacytosis through prospective analysis of autoimmune patients

**Figure 2:**
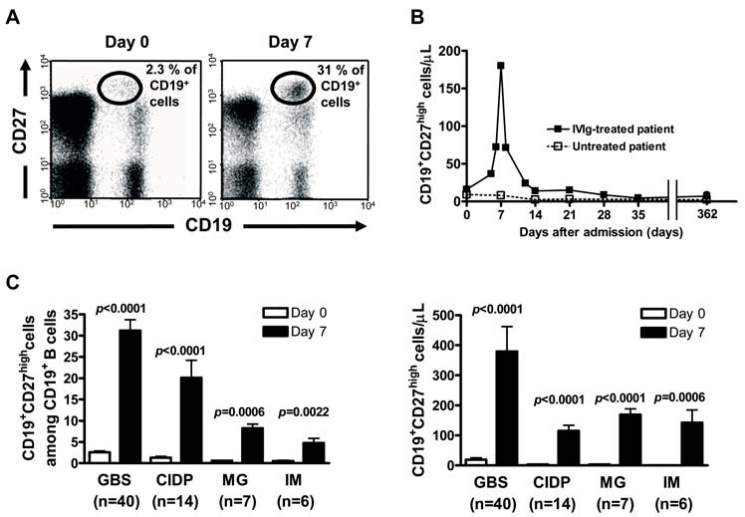
Prospective analysis of plasmacytosis in autoimmune patients. (A) Cytometric analysis of circulating CD19^+^CD27^high^ plasma cells on day 0 and day 7 of IVIg treatment in a representative GBS patient. Proportions of CD19^+^CD27^high^ plasma cells among CD19^+^ cells are indicated. (B) Peripheral CD19^+^CD27^high^ plasma cell kinetics in representative GBS patients. (C) Proportions among CD19^+^ cells (left panel) and absolute numbers (right panel) of IVIg-mobilized CD19^+^CD27^high^ plasma cells. GBS (disability grade 3 or more, n = 40) and CIDP (n = 14) patients received IVIg only, whereas MG (n = 7) and IM (n = 6) patients received IVIg and steroids. Data are mean ± s.e.m. Mean values were compared using Mann-Whitney U test.

We next examined if the data gathered during our retrospective analysis could be confirmed in a consecutive population of GBS, CIDP, MG and IM patients. Non-ambulant GBS patients and CIDP patients were treated with IVIg, whereas MG and IM patients received IVIg and steroids. Using flow cytometry, high CD27 expression on CD19^+^ B cells allows us to distinguish plasma cells from other CD19^+^ B cells (Figure 2A) [13], [14]. A longitudinal analysis of these subpopulations was performed just prior to the start of treatment with IVIg and every 7 days during 1 month (Figure 2B). Compared to baseline values (day 0), one finds on the 7^th^ day a very significant mobilization of circulating CD19^+^CD27^high^ plasma cells in IVIg-treated patients (Figure 2). Importantly, IVIg-mobilized CD19^+^CD27^high^ cell counts strongly correlated with plasma cell counts measured by blood smears (r = 0.94, *p*<0.0001, n = 67, data not shown), indicating that the same type of plasma cell was indeed monitored at day 7 of IVIg in both retrospective and prospective studies.

In comparison, levels of absolute CD19^+^CD27^high^ plasma cell counts remained unchanged during the first week following admission in ambulant GBS patients without any specific treatment (5.9 ± 2.4 cells/µl vs 6.1 ± 4.2 cells/µl, n = 10, *p* = 0.53, data not shown).

### Mobilization of immature antibody secreting cells 7 days post-IVIg

The circulating plasma cells we observed could correspond to recently generated and/or highly differentiated plasma cells. Recently, downregulation of HLA-DR [15], and upregulation of CD138 [16] and CXCR4 [17], [18] have been shown to be characteristic features of mature plasma cells exiting from the bone marrow. We first verified that the IVIg-mobilized CD19^+^CD27^high^ cells contained IgG, and co-expressed CD38 and CD138 (Figure S1A). In addition, their numbers were clearly proportional to the number of cells capable of secreting IgG antibodies *in vitro* in response to a non-specific stimulus (r = 0.99, *p* = 0.0028, n = 6; Figure S1B and Methods S1).

**Figure 3:**
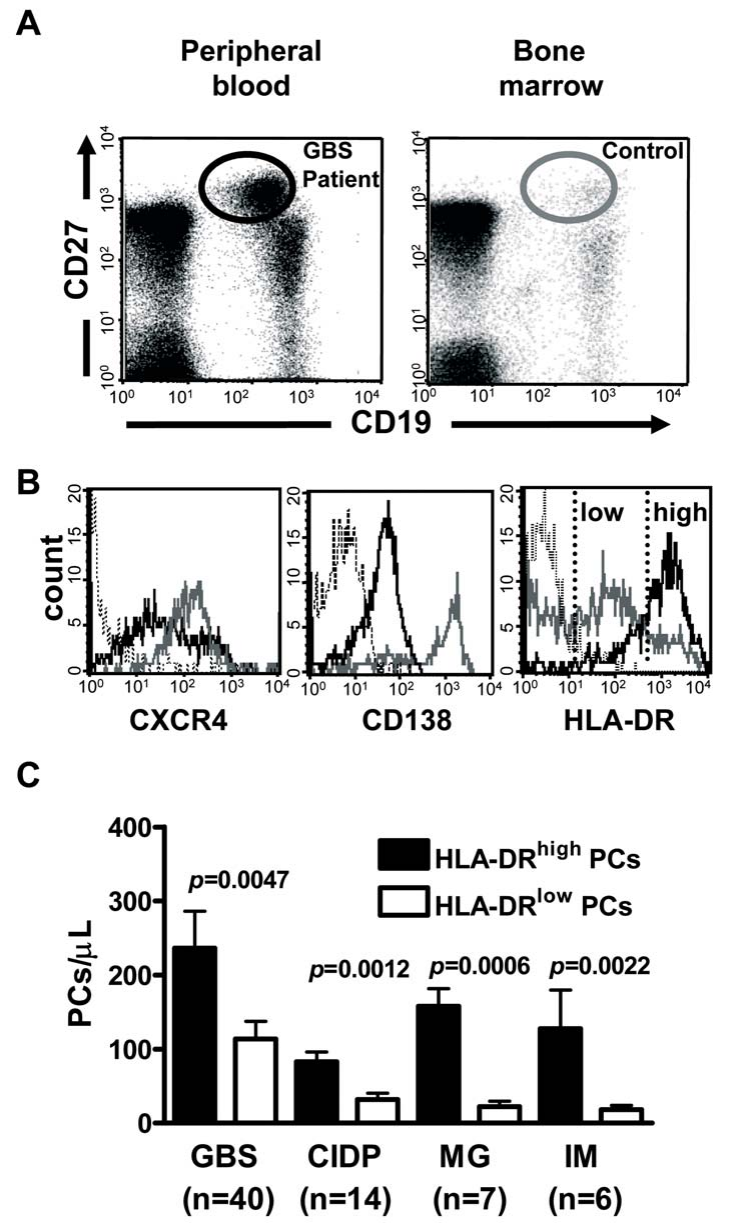
Most IVIg-mobilized CD19^+^CD27^high^ cells are immature. (A) Representative analyses of circulating and bone marrow CD19^+^CD27^high^ cells in a GBS patient 7 days after IVIg infusion and in a healthy bone marrow donor. (B) Control mature medullary CD19^+^CD27^high^ plasma cells (as identified in panel A, right) were CXCR4^high^CD138^high^HLA-DR^low^ (gray line). Circulating IVIg-mobilized CD19^+^CD27^high^ plasma cells were CXCR4^low^CD138^low^HLA-DR^high^ (solid line). Isotype controls were presented (dotted line). (C) Balance between immature (HLA-DR^high^) and mature (HLA-DR^low^) plasma cells (PCs) in IVIg-treated patients 7 days after treatment. GBS (disability grade 3 or more, n = 40) and CIDP (n = 14) patients received IVIg only, whereas MG (n = 7) and IM (n = 6) patients received IVIg and steroids. Data are mean ± s.e.m. Comparisons of indicated mean values were performed by use of Mann-Whitney U test.

We next revealed that the majority of IVIg-mobilized plasma cells were CXCR4^low^, CD138^low^, and HLA-DR^high^, and as such, relatively immature (Figure 3A left panel, B-C). Here, medullary cells from a healthy control were taken as reference in order to present CXCR4, CD138, and HLA-DR levels, typically associated with terminal differentiation into mature plasma cells in the bone marrow (Figure 3A, right panel).

**Figure 4:**
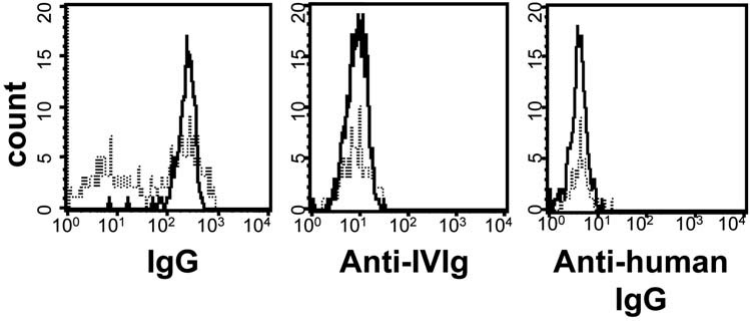
IVIg-mobilized plasma cells are neither IVIg- nor human IgG-specific. CD19^+^CD27^high^ plasma cells were analyzed following intracellular staining with anti-IgG FITC, IVIg FITC and human IgG FITC before (dotted line) and 7 days after IVIg infusion (black line). One representative individual out of 17 analyzed.

We further clarified that plasma-cell mobilization was not the mere consequence of an immunization against IVIg. PBMCs were stained *in vitro* with fluorescently labeled IVIg or purified human IgG. As shown, mean fluorescence levels measured on CD19^+^CD27^high^ cells before treatment remained unchanged 7 days after IVIg infusion (Figure 4 middle and right panels).

### Clinical evolution in relation to day 7 plasmacytosis in GBS

**Figure 5:**
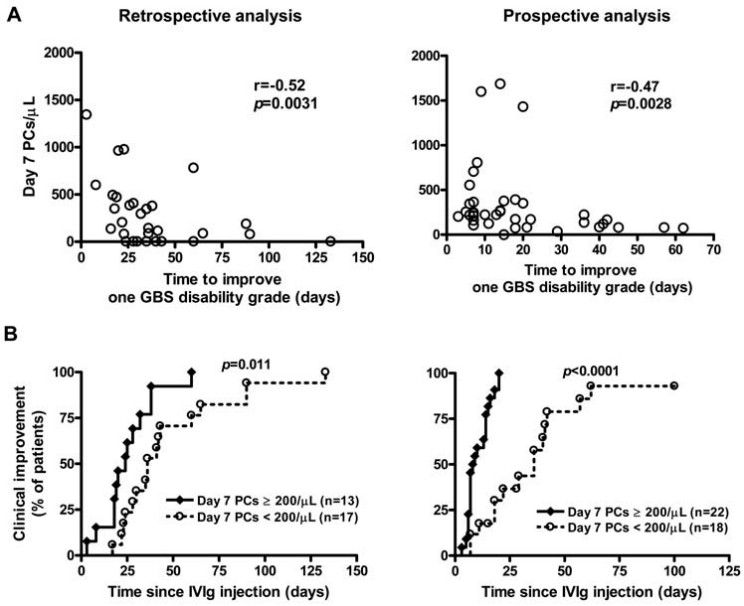
Correlation between the number of plasma cells and clinical improvement in retrospective (n = 30, left panels) and prospective (n = 40, right panels) studies. (A) Strong negative correlation in both studies between the absolute number of IVIg-mobilized plasma cells (PCs) and time to improve one GBS disability-grade. Correlation was analyzed using Spearman test. (B) Day 7 plasmacytosis correlates with faster clinical improvement. Kaplan-Meier analysis of time to one disability-grade improvement following IVIg in relation to day 7 plasma cell counts enumerated on a routine hematology analyzer and confirmed by blood smear exam.

Finally, we sought to determine if IVIg-related plasmacytosis could serve as a marker of effectiveness of this treatment. Firstly, we plotted plasma cell counts 7 days post-IVIg and time to improvement of one GBS disability-grade, in order to assess the possible correlation between the number of IVIg-mobilized plasma cells and clinical improvement (Figure 5A). Retrospective analysis was limited to severely disabled patients in order to avoid bias related to potential deficiencies in minor symptom recording. We found a strong negative correlation between the absolute number of IVIg-mobilized plasma cells and time to improve one GBS disability-grade in both retrospective and prospective studies (r = −0.52, *p* = 0.0031, n = 30, r = −0.47, *p* = 0.0028, n = 40, respectively; Figure 5A).

Secondly, GBS patients were divided into two groups depending on whether they had or had not shown a significant plasmacytosis (≥ 200 plasma cells/µL) on the 7^th^ day of treatment. Prominent plasmacytosis was noted in 12 out of 30 patients and 22 out of 40 severely disabled patients in retrospective and prospective analyses, respectively. Importantly, the two groups did not differ in any clinical prognostic factors, including intervals between motor deficit onset and treatment initiation (*p* = 0.27, *p* = 0.26, respectively), age (*p* = 0.93, *p* = 0.98, respectively), preceding diarrhoea (*p*>0.99, *p* = 0.51, respectively), and rapid disease onset (*p* = 0.44, *p* = 0.40, respectively). Patients having shown a plasmacytosis greater than or equal to 200 plasma cells/µl on the 7^th^ day of treatment recovered faster than those who had not shown such an expansion (*p* = 0.011, *p*<0.0001, respectively; Figure 5B). More patients having presented with a prominent IVIg-related plasmacytosis achieved independent walking at day 56, compared with the others (*p* = 0.0069, *p* = 0.0095, respectively).

A more detailed analysis of the consecutive population revealed that patients exhibiting more than 200 IVIg-mobilized plasma cells/µl showed less severe GBS disability-grade at day 14 (3.1 ± 0.94 vs 4.2 ± 1.1, *p* = 0.0031, data not shown). In addition, the patient group with a prominent IVIg-related plasmacytosis less frequently required mechanical ventilation (*p* = 0.0073) and for shorter time than the group not showing IVIg-related plasmacytosis (18 ± 4.0 days, n = 3 vs 39 ± 16 days, n = 10, *p* = 0.0034, data not shown).

## Discussion

Here we show that a prominent plasmacytosis was very frequently associated with administration of IVIg in autoimmune patients. More importantly, the level of circulating plasma cells on the 7^th^ day of IVIg treatment correlated with clinical improvement in patients with GBS. These findings indicate that plasma cell count on the 7^th^ day of IVIg will be useful to distinguish patients that have beneficial IVIg effects from those who would need more individualized treatment, and to reconsider the treatment regimen during the acute phase of GBS in the latter.

Low levels of circulating CD19^+^CD27^high^ plasma cells were occasionally detected in healthy individuals (5.2 ± 2.4 cells/µl, n = 22, data not shown) or in untreated autoimmune patients in our own study (Figure 2C). A fugacious plasmacytosis is in fact a well known physiological consequence of any recent antigenic stimulation [15], [19], [20], but typically much less than 100 lymphoplasmocytoid cells can be found in one microliter of blood in such occasions, even in autoimmune patients with active disease flares [14]. Here, we verified that a prominent plasmacytosis was not a disease- or an infectious-related event. We also showed that IVIg-mobilized plasma cells were not IVIg-specific (Figure 4 middle panel), and therefore did not simply reflect an immunization against the infused products.

A very significant proportion of CD19^+^CD27^high^ plasma cells on the 7^th^ day of IVIg were detected in all GBS patients, as well as in other autoimmune patients (Figure 2C). GBS patients presented with significantly more day 7 plasma cells (31.2 ± 16.1% among CD19^+^ B cells, n = 40), compared with other autoimmune patients (CIDP; 20.1 ± 15.4%, n = 14, *p* = 0.0092, MG; 8.3 ± 2.9%, n = 7, *p*<0.0001, IM; 5.3 ± 2.7%, n = 6, *p*<0.0001; Figure 2C left panel). The less prominent plasma-cell mobilization in MG and IM patients could be related to the fact that these patients received steroids either before or during IVIg infusion. However, GBS and CIDP groups were homogenous in terms of age, sex and treatment received. Therefore, we can only postulate that IVIg-related plasmacytosis would be more pronounced in the context of an ongoing immune response, compared to chronic conditions such as CIDP. In order to test that hypothesis, IVIg-related plasmacytosis should be measured in healthy controls.

In GBS patients, the magnitude of IVIg-related plasmacytosis correlated with improvement of neurological function, including achievement of independent locomotion and discontinuation of mechanical ventilation. Importantly, clinical prognostic factors were demonstrated to be homogenous between patients with a prominent IVIg-related plasmacytosis and the others, indicating that IVIg-mobilized plasma cells could be served as a biological prognostic marker. Recently, a prognostic scoring system for GBS has been reported to predict the capacity of independent locomotion at 6 months [5]. This scoring system is based on age, previous episode of diarrhoea and GBS disability-grade at 2 weeks of treatment. We evaluated how day 7 plasmacytosis performs, compared with this score (Erasmus GBS outcome score). In the consecutive population we studied, we show that the group of patients with a prominent IVIg-related plasmacytosis will recover faster than the others according to classic criteria (ventilation time, ability to walk independently at 2 months and 6 months). We show that the Erasmus GBS outcome score in that group could also have predicted a more favourable evolution, compared with the other (*p* = 0.0018). As stated above, retrospective analysis was limited to severely disabled patients in order to avoid bias related to minor symptom recording. In that retrospective population-based study, we also show that the group of patients with a prominent IVIg-related plasmacytosis will recover faster than the other. Interestingly, we found that the Erasmus GBS outcome score would not have predicted a more favourable evolution in that group (*p* = 0.55). Therefore, plasma-cell monitoring appears to have an added value over the Erasmus GBS outcome score for the prognostic evaluation of severe GBS patients.

Further observations in the 5 GBS patients who relapsed within 28 days after IVIg give further comfort to the prognostic value of day 7 plasmacytosis. These patients had significantly less circulating day 7 plasma cells, compared with the others (68 ± 72 cells/µl, n = 5 vs 369 ± 426 cells/µl, n = 35, *p* = 0.0079), and were treated with several courses of IVIg. Despite repeating IVIg treatment, 2 such refractory patients died 28 days and 100 days after the first IVIg course. It is therefore unlikely that repeating IVIg treatment in refractory patients will be adequate. Cost would be reduced, and treatment could be optimized by defining which patients would benefit from an alternative treatment instead of receiving several IVIg courses. However, more patients need to be studied before we can recommend not repeating IVIg infusion in the absence of plasmacytosis after the first IVIg course.

The mobilization of a large number of plasma cells concurrently with clinical improvement might seem surprising. The detection of these cells is rather typically associated with autoimmune disease flares, for instance in multiple sclerosis and systemic lupus erythematosus [14], [21]. Our data, as counter-intuitive as they may seem, are nevertheless in good agreement with proposed models of plasma-cell dynamics during humoral immune responses [15]. According to the later, newly generated plasmablasts enter into competition with older cells for their medullary niches. Similarly, we would like to propose that following IVIg treatment, a large “wave” of mobilized cells induces a significant renewal of the repertoire of antibody-secreting cells. A “re-equilibration” of the repertoire of circulating antibodies in favour of non-pathogenic antibodies could then ensue. As stated above, it is strongly suspected that antibody-secreting plasma cells which cross-react with nervous structures are induced during a preceding infectious episodes [22], and reside in the survival niches of the bone marrow in patients with GBS [23]. One can imagine that a proportion of these cells, among others, would be chased from the marrow by a wave of new polyclonal immigrants [15], [24]. Given the long half-life of antibodies, this mechanism may be difficult to reconcile with the rapid mode of action of IVIg. Other mechanisms have been proposed to account for the anti-inflammatory effect of IVIg [25], [26]. It is now generally accepted that IVIg has multiple therapeutic effects that would act in concert [27]. IVIg could, for instance, directly influence plasma-cell survival [28], [29]. Plasma-cell mobilization could nevertheless have a long lasting impact on the pool of long-lived plasma cells that are not affected by classic immunomodulation treatments [30]. This particular mode of action of IVIg could explain its superiority over steroids in certain indications such as GBS [12], [31], [32]. Interestingly, it is notable that Pemphigus Vulgaris patients who do not respond to steroids plus immunosuppressive agents can nevertheless respond to IVIg [33].

IVIg promotes immature plasma-cell mobilization in patients with GBS, CIDP, MG and IM (Figure 3C). Even if it has been demonstrated that IVIg could accelerate the *in vitro* maturation of B lymphocytes [34], the origin of the mobilized plasma cells [35] and the mechanism through which this treatment induces their mobilization remain unclear. It will therefore be interesting to determine how IVIg is able to influence the maturation, the migration, and ultimately the replenishment of plasma cells.

In spite of IVIg treatment, approximately 20% of the patients with GBS are unable to walk without assistance after 1 year [6], [7]. Day 7 plasma-cell monitoring will be helpful to predict which patients will improve after IVIg treatment, and therefore will prompt the early implementation of more individualized therapeutic strategies in such patients at a greater risk of poor outcome.

## Supporting Information

Figure S1 IVIg-mobilized CD19^+^CD27^high^ plasma cells were IgG-secreting cells. (A) A representative 4-colour cytofluorometric analysis of GBS PBMCs 7 days after initiation of IVIg infusion. Intracellular analysis revealed that post-IVIg CD19^+^CD27^high^ plasma cells (gated as indicated) were intracellular IgG^+^IgM^−^ and membrane CD38^+^CD138^+^ (insets). (B) Correlation between flow cytometry-enumerated CD19^+^CD27^high^ cells and ELISPOT-enumerated IgG-secreting cells (SCs) in 6 GBS patients treated with IVIg. Cytofluorometric analysis and ELISPOT assay were simultaneously performed 7 days post-IVIg infusion. Correlation was analyzed using Spearman test.
(10.10 MB TIF)

Methods S1 (0.03 MB DOC)
