# Supplementary material for: Prominent Plasmacytosis Following Intravenous Immunoglobulin Correlates with Clinical Improvement in Guillain-Barré Syndrome
Source: PLoS One. 2008 May 7;3(5):e2109. doi: 10.1371/journal.pone.0002109 (PMC2362102; doi:10.1371/journal.pone.0002109)
Supplement: Methods S1 — (0.03 MB DOC) [file pone.0002109.s002.doc]

# Supplementary materials and methods

**Enumeration of IgG-secreting cells by ELISPOT**

PBMCs purified over Ficoll (Amersham Pharmacia Biotech, Uppsala, Sweden) were enriched for B lymphocytes using anti-CD19 micromagnetic beads (Miltenyi-Biotech, Paris, France). ELISPOT 96-well filter plates (MAHA N4510, Millipore, Saint Quentin-en-Yvelines, France) were coated with goat anti-human immunoglobulin (Ig) (Sigma). Plates were washed and then blocked with PBS/5% BSA for 2 hours at 37°C. Triplicate wells containing 50 to 75000 PBMCs were incubated in RPMI-1640 supplemented with 10% fetal calf serum at 37°C and 5% CO_2_ for 5 hours. Negative control wells lacked cells or Ig coating. Plates were then washed with PBS/Tween and incubated overnight at 4°C in 1 μg/ml mouse anti-human IgG- or IgM-biotin conjugated antibody (Sigma) in PBS/0.5% BSA. Plates were again washed with PBS/Tween and then developed using FAST BCIP/NBT Substrate System (Sigma). Only wells that contained less than 100 immunospots were taken into account. Spots of 0.05 to 0.2 mm circular *foci*, densely granulated with a colour decrease from the centre to the periphery, were counted using an automated plate reader (Carl Zeiss Vision, Germany).
